# Incidence, risk factors and maternal outcomes of unsuspected placenta accreta spectrum disorders: a retrospective cohort study

**DOI:** 10.1186/s12884-024-06254-z

**Published:** 2024-01-23

**Authors:** Jianlin Zhao, Qin Li, E Liao, Haijun Shi, Xin Luo, Lan Zhang, Hongbo Qi, Hua Zhang, Junnan Li

**Affiliations:** 1 The Department of Obstetrics, The First Affiliated Hospital of Chongqing Medical University, Chongqing, 400016 China; 2 Chongqing Key Laboratory of Maternal and Fetal Medicine, Chongqing Medical University, Chongqing, 400016 China; 3 Department of Obstetrics and Gynecology, Chongqing Health Center for Women and Children, Women and Children’s Hospital of Chongqing Medical University, Chongqing, 401147 China; 4 Department of Obstetrics and Gynecology, Maternal and Child Hospital of Hubei Province, Tongji Medical College, Huazhong University of Science and Technology, Wuhan, 430070 Hubei Province China

**Keywords:** Unsuspected placenta accreta spectrum, Incidence, Risk factors, Maternal outcomes

## Abstract

**Background:**

To identify incidence and underlying risk factors for unsuspected placenta accreta spectrum (PAS) and compare the maternal outcomes between suspected and unsuspected cases in three large academic referral centers.

**Methods:**

A retrospective cohort study was conducted in three university-based tertiary referral centers from Jan 1st, 2013, to Dec 31st, 2022. All cases of PAS confirmed by pathology were included in the study. Unsuspected PAS cases were diagnosed at the time of delivery, while suspected cases served as the control group. Potential risk factors were compared between the two groups. Multivariable regression model was also performed to identify risk factors. Maternal outcomes were also evaluated.

**Results:**

A total of 339 pathology-confirmed PAS cases were included in the study out of 415,470 deliveries, of which 35.4% (*n* = 120) were unsuspected cases. Unsuspected PAS cases were 7.9 times more likely to have a history of intrauterine adhesions (adjusted odds ratio [aOR] 7.93; 95% confidence interval [CI] 2.35–26.81), 7.0 times more likely to have a history of clinically confirmed PAS (aOR, 6.99; 95% CI 2.85–17.18), 6.3 times more likely to have a posterior placenta (aOR, 6.30; 95% CI 3.48–11.40), and 3.4 times more likely to have a history of placenta previa (aOR, 3.41; 95% CI 1.18–9.82). On the other hand, cases with gravidity > 3, placenta previa, and/or a history of previous cesarean delivery were more likely to be diagnosed antenatally (aOR 0.40, 0.19, 0.36; 95% CI 0.22–0.74, 0.09–0.40, 0.19–0.70). Although the suspected PAS group had a higher proportion of invasive cases and abdominal and pelvic organ injuries (74.4% vs. 25.8%, *p* < 0.001; 6.8% vs. 1.7%, *p* = 0.037), the maternal outcomes were more favorable in the sPAS group, with a lower median volume of 24-hour blood loss and blood product transfusion (estimated blood loss in 24 h, 1000 [800–2000] vs. 2000 [1400–2400], *p* < 0.001; RBC unit transfusion, 0 [0-800] vs. 800 [600–1000], *p* < 0.001; fresh-frozen plasma transfusion, 0 [0-450] vs. 600 [400–800], *p* < 0.001).

**Conclusions:**

Our findings indicate that 35% of patients with PAS were unsuspected prior to delivery. Factors associated with PAS being unsuspected prior to delivery include a history of intrauterine adhesions, a history of clinically confirmed PAS, a posterior placenta, and a history of placenta previa. Additionally, gravidity > 3, a history of previous cesarean delivery, and placenta previa increase the likelihood of antenatal diagnosis.

**Supplementary Information:**

The online version contains supplementary material available at 10.1186/s12884-024-06254-z.

## Introduction

Placenta accreta spectrum (PAS) disorder refers to excessive placental invasion into myometrium, which is known to be associated with serious obstetric complications [1]. The incidence of PAS has increased dramatically in recent years due to the rise in global cesarean delivery rates, particularly in countries like China where the rate has exceeded 50% [2, 3]. Several studies have shown that PAS, especially severe cases of PAS, such as increta or percreta, are known to be associated with major hemorrhage, peripartum hysterectomy, and potentially complicated surgeries. Timely antenatal suspicion and specialized care in experienced maternal-fetal medical centers can help reduce the morbidity and mortality associated with PAS [4]. Therefore, identifying PAS antenatally is of paramount importance.

Prenatal suspicion of PAS mainly relies on experienced clinicians’ recognition of high-risk factors and then detailed ultrasonography based on high-risk clinical factors, resulting in varying prenatal suspicion rates of PAS among different studies, and these studies have reached inconsistent conclusions regarding maternal outcomes [5–7]. Furthermore, few studies reported the differences in the clinical characteristics between the suspected cases (sPAS) and unsuspected cases (uPAS). And most importantly, there are no relevant studies on which factors may affect the prenatal recognition of PAS based on these clinical differences. Therefore, we collected data on all histology-confirmed PAS cases in three university-based referral centers in China from 2013 to 2022, hoping to answer these questions.

Our primary objective was to preliminary determine the rate of uPAS in China and to identify risk factors that are associated with uPAS. The secondary objective was to compare differences in clinical characteristics and maternal outcomes between the two groups. Identification of uPAS-related factors can lead to future-oriented quality improvement initiatives to optimize management of this condition and improve maternal outcomes.

## Methods

### Study design and population

A retrospective cohort study was performed in three university-based tertiary referral centers from Jan 1st, 2013, to Dec 31st, 2022. To identify all cases, data were extracted from the electronic health record. Inclusion criteria consisted of patients who met the following three criteria:1) had a delivery at 1 of the 3 hospitals; 2) had a hysterectomy performed at the delivery; 3) had a pathology-confirmed diagnosis of PAS. Twin pregnancies were included in this study.

Antepartum PAS suspicion was based on any PAS ultrasound imaging modalities relying on antenatal diagnosis of the experienced ultrasonographers or maternal fetal medicine specialist. The specific ultrasound signs were not retrospectively reviewed for this study, as the focus was on identifying risk factors and outcomes rather than specific ultrasound characteristics. For patients suspected with PAS, they would be referred to PAS-experienced obstetricians. Clinical backgrounds, ultrasound, and/or MRI imaging would all be taken into account to determine the severity of PAS, and after consultation, a consensual management plan would be given on a case-by-case basis and with full considerations of maternal willingness to determine: (1) whether placement of abdominal aortic balloon was needed before cesarean delivery; (2) whether uterus should be preserved in surgery; (3) delivery time and mode of delivery would be planned. For pregnant women who were not suspected for PAS, subsequent prenatal management would be carried out as guideline [8].

### Data collection and definition

Based on previous studies [5, 9–12], detailed demographic data, and established and potential risk factors such as maternal age, prepregnancy BMI, gravidity and parity, pregnancy complications, multiple gestation, in vitro fertilization, placenta previa, placental location (anterior, posterior, bilateral and fundus), adenomyosis/current fibroids, mullerian anomaly, and PAS-related history, were included. Other basic characteristics such as delivery mode and delivery weeks were also recorded. Maternal outcomes mainly included estimated blood loss in 24 h, blood products transfusion, abdominal aorta balloon block, abdominal and pelvic organ injuries, ICU admission and postoperative hospital stay. All the data were double-checked by trained research personnel. We categorized PAS diagnoses into noninvasive (accreta) or invasive (increta or percreta) since available data showed lower morbidity for placenta accreta and similar morbidity for the two invasive forms [13, 14].

Clinically confirmed PAS was defined with one of the following criteria: (1) inability to fully remove the placenta manually, despite active management of the third stage of labor, leading to evidence of placental retention; (2) presence of sonographic evidence of retained placental fragments requiring removal after vaginal delivery; (3) experiencing heavy bleeding from the implantation site after placental removal during cesarean delivery, but the uterus could be preserved. We consider a history of clinically confirmed PAS if cases had clinically confirmed PAS in previous pregnancies.

### Statistical analysis

Descriptive statistics (mean ± standard deviation for normally distributed continuous variables and median and interquartile range for non-normally distributed continuous variables; frequency and percentage for categorical variables) were calculated for each group. All continuous variables were tested for normality using the Kolmogorov-Smirnov test and visual plots, such as histograms and Q-Q plots. For normally distributed variables, two-sample t-test was used. For nonnormally distributed continuous variables, the Wilcoxon rank-sum test was used. Categorical data were evaluated with the x^2^ test or Fisher’s exact test when the suspected cell count was < 5. Logistic regression was used to adjust for multiple variables. To compare PAS risk factors between the uPAS and sPAS groups, all potential factors with a univariate *P*-value of 0.25 or less were included in the multi-variable model. Demographic and obstetric characteristics that were significantly associated with PAS were also evaluated as confounders in this association. Result was considered statistically significant at the *P* < 0.05 level of significance. All analyses were performed with SPSS software (IBM SPSS Statistics 26, Armonk, NY, USA).

## Results

### General characteristics

There were 415,470 deliveries in ten years period at the three hospitals. After reviewing pathology reports, 339 patients were diagnosed with placenta accreta spectrum (PAS), and 35.4% (*n* = 120) of the PAS cases were not suspected antenatally. Table S1 provides an overview of the annual number of PAS cases, suspected PAS cases (uPAS), and unsuspected PAS cases (sPAS), as well as the prenatal diagnostic rate of PAS across the three hospitals. Table 1 compares the basic characteristics and potential risk factors between two groups, considering current conditions and the medical history related to PAS. Compared to sPAS group, the proportion of gravidity > 3 was lower (43.3% vs. 62.6%) in the uPAS group, and the proportion of nulliparous women was higher (8.3% vs. 2.7%). Although both groups had a high prevalence of placenta previa, the uPAS group had a relatively lower proportion (70.8% vs. 92.7%). Notably, In the uPAS group, more than half of the placentas (65%) were located in the posterior wall, whereas in the sPAS group, more than half (66.2%) were located in the anterior wall. Additionally, the sPAS group had a significantly higher proportion of invasive PAS (increta or percreta) compared to the uPAS group (74.4% vs. 25.8%). Regarding the delivery mode, nearly all pregnant women underwent cesarean delivery (CD). However, it is worth noting that 99.1% of cases in the sPAS group had a planned CD, while the corresponding figure in the uPAS group was 67.5%. Furthermore, women in the sPAS group delivered at an earlier gestational age compared to those in the uPAS group [35.0 weeks (34.2–35.5) vs. 37.4 weeks (35.5–39.1)].

**Table 1:** Basic characteristics and potential risk factors of the study participants in the two study groups

Characteristics and potential risk factors	sPAS(219)	uPAS(120)	*p* value
**Present conditions**			
Advanced maternal age	59(26.9)	27(22.5)	0.369^a^
Pre-pregnancy BMI	21.5 ± 3.2	21.2 ± 2.5	0.326^b^
Gravidity	4(3–5)	3(2–5)	0.004^c^
Gravidity > 3	137(62.6)	52(43.3)	< 0.001^a^
Parity	1(1–2)	1(1–2)	0.122^c^
Parity > 1	76(34.7)	35(29.2)	0.299^a^
Nulliparous women	6(2.7)	10(8.3)	0.020^a^
Multiple gestation	10(4.6)	8(6.7)	0.409^a^
In vitro fertilization	9(4.1)	8(6.7)	0.302^a^
Placenta previa	203(92.7)	85(70.8)	< 0.001^a^
Placenta location			< 0.001^a^
* Anterior*	145(66.2)	34(28.3)	
* Posterior*	57(26.0)	78(65.0)	
* bilateral/fundal*	17(7.8)	8(6.7)	
Pregnancy complications	35(16.0)	11(9.2)	0.080^a^
Adenomyosis/current fibroids	10(4.6)	6(5.0)	0.857^a^
Mullerian anomaly	3(1.4)	3(2.5)	0.670^d^
Severity of invasion			< 0.001^a^
* Noninvasive (accreta)*	56(25.6)	89(74.2)	
* Invasive (increta/percreta)*	163(74.4)	31(25.8)	
Delivery mode			< 0.001^d^
* Vaginal/operative vaginal*	0(0)	4(3.3) ^e^	
* Planed CD*	217(99.1)	81(67.5)	
* Emergency CD*	2(0.9)	35(29.2)	
Delivery gestational weeks	35.0(34.2–35.5)	37.4(35.5–39.1)	< 0.001^c^
**Previous conditions**			
History of previous CD	189(86.3)	79(65.8)	< 0.001^a^
History of clinical confirmed PAS	14(6.4)	25(20.8)	< 0.001^a^
History of endometrial ablation	1(0.5)	2(1.7)	0.287^d^
Previous uterine artery embolization for PPH	7(3.2)	6(5.0)	0.395^d^
History of myomectomy	0(0.0)	2(0.9)	NA
History of operative hysteroscopy	8(3.7)	10(8.3)	0.066^a^
History of intrauterine adhesions (IUA)	7(3.2)	13(10.8)	0.004^a^
Dilation and evacuation of the uterus	17(7.8)	12(10.0)	0.481^a^
History of placenta previa	11(5.0)	11(9.2)	0.139^a^
Previous manual removal of placenta/placenta retention	8(3.7)	12(10.0)	0.018^a^

Data are presented as mean ± standard deviation or median (interquartile range) or n (percentage)

BMI, body mass index; CD, Cesarean delivery; PAS, placenta accreta spectrum; PPH, postpartum haemorrhage; sPAS, suspected placenta accreta spectrum before delivery; uPAS, unsuspected placenta accreta spectrum before delivery

^a^ Chi-square test; ^b^ Two-sample t-test; ^c^ Wilcoxon rank-sum test; ^d^ Fisher exact test

^e^ All these cases were performed hysterectomy after vaginal delivery

Regarding historical data related to PAS, 86.3% of women had a previous history of CD in the sPAS group, whereas this percentage was slightly lower at 65.8% in the uPAS group. Notably, the rate of previous clinical confirmed PAS was significantly higher in the uPAS group compared to the sPAS group (20.8% vs. 6.4%). Additionally, the uPAS group exhibited higher percentages of cases with a history of intrauterine adhesions and previous manual removal of placenta/placenta retention (10.8% vs. 3.2% and 10% vs. 3.7%, respectively). Although these proportions were not high in either group, the differences were statistically significant.

### Screening for risk factors by logistic analysis

All potential risk factors with *P* < 0.25 in Table 1 were included in the logistic regression model, and the results with significant variables presented with corresponding odds ratios are shown in Table 2. Pregnant women in the uPAS group had significantly higher odds of having a history of intrauterine adhesions (adjusted odds ratio [aOR] 7.93, 95% CI 2.35–26.81), clinical confirmed PAS (aOR 6.99, 95% CI 2.85–17.18) and placenta previa (aOR 3.41, 95% CI 1.18–9.82) compared to the sPAS group. Placenta location was also associated with uPAS, with posterior placenta having 6.30 times the odds of being uPAS (aOR 6.30, 95% CI 3.48–11.40) compared to anterior placenta. However, our results also indicated that PAS cases with gravidity > 3, history of previous CD and/or placenta previa were more likely to be diagnosed antenatally (gravidity > 3, aOR, 0.40; 95% CI 0.22–0.74; history of previous CD, aOR, 0.36; 95% CI 0.19–0.70; placenta previa, aOR, 0.19; 95% CI 0.09–0.40).

**Table 2:** Risk factors associated with uPAS

Factors	Odds ratio (95% confidence interval)	
	Unadjusted^a^	Adjusted^b^
History of intrauterine adhesions	3.68(1.43–9.49)	7.93(2.35–26.81)
History of clinical confirmed PAS	3.85(1.92–7.74)	6.99(2.85–17.18)
Placenta location		
* Anterior(ref)*	NA	NA
* Posterior*	5.84(3.52–9.68)	6.30(3.48–11.40)
* bilateral/fundal*	2.01(0.80–5.03)	2.64(0.90–7.69) ^c^
History of placenta previa	1.91(0.80–4.54)	3.41(1.18–9.82)
Gravidity > 3	0.46(0.29–0.72)	0.40(0.22–0.74)
History of previous CD	0.31(0.18–0.52)	0.36(0.19–0.70)
Placenta previa	0.19(0.10–0.36)	0.19(0.09–0.40)

^a^ Data are from univariate analysis; ^b^ Data are from the multiple logistic regression model; ^c^*p* = 0.076

PAS, placenta accreta spectrum

**Fig. 1:**
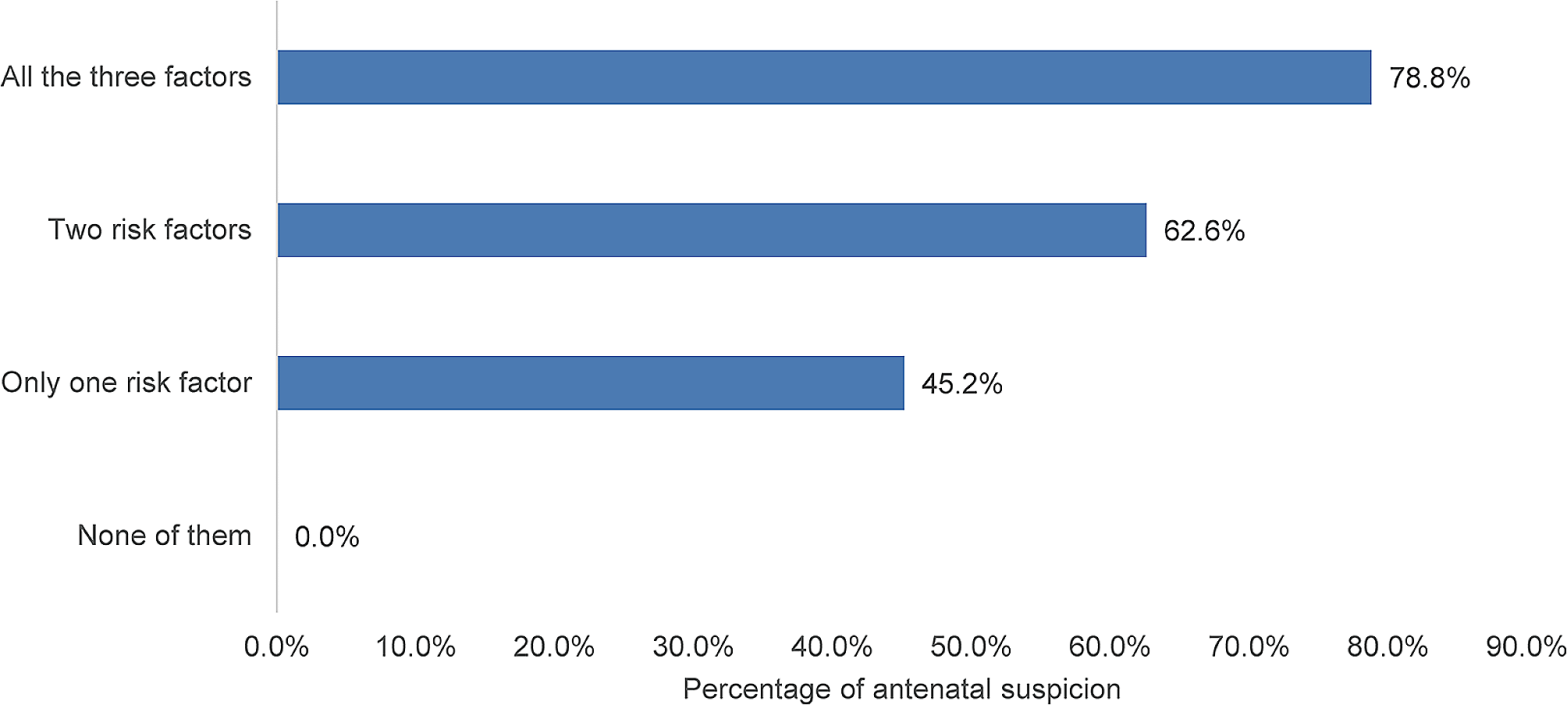
Rate of suspicion classified by the number of major known risk factors

Considering that multiple gravidities (gravidity > 3), a history of previous CD (number of CD ≥ 1), and/or placenta previa are established risk factors for PAS, we divided the cases into four groups based on the number of these factors. Figure 1 illustrates the percentage of antenatal suspicion among these four groups. None of the PAS cases were suspected antenatally when none of these factors were present, while 78.8% of cases were suspected antenatally when all three risk factors were present. The suspicion rates of PAS were 62.6% and 45.2% when two or only one risk factor was present, respectively. More detailed data related to this figure can be found in the supplementary material (Table S2).

### Comparison of maternal outcomes

Table 3 showed the maternal outcomes of the two groups. In terms of preoperative procedure, 30.1% of sPAS women placed abdominal aorta balloon before surgery, while none of uPAS women had this procedure. The incidence of abdominal and pelvic organ injuries was higher in the sPAS group compared to the uPAS group (6.8% vs. 1.7%). Regarding 24-hour postpartum blood loss, the sPAS group was significantly lower [1000 (800–2000) vs. 2000 (1400–2400)], and correspondingly, the infusion of blood products (red blood cell and fresh-frozen plasma) was also statistically lower in the sPAS group [0(0-800) vs. 800(600–1000); 0(0-450) vs. 600(400–800)]. The length of stay after CD were longer in the sPAS group [8(7–9) vs. 7(7–8)].

**Table 3:** Maternal outcomes in the two study groups

Variables	sPAS(219)	uPAS(120)	*p* value
Abdominal aorta balloon block	66(30.1)	0(0)	NA
Abdominal and Pelvic organ injuries	15(6.8)	2(1.7)	0.037^a^
* Bladder injury*	12(5.5)	2(1.7)	
* Vascular injury*	2(0.9)	0(0)	
* Intestinal injury*	1(0.4)	0(0)	
Estimated blood loss in 24 h	1000(800–2000)	2000(1400–2400)	< 0.001^b^
RBC transfusion	0(0-800)	800(600–1000)	< 0.001^b^
Fresh-frozen plasma transfusion	0(0-450)	600(400–800)	< 0.001^b^
Cryoprecipitation	0(0–0)	0(0–0)	NA
Platelet transfusion	0(0–0)	0(0–0)	NA
ICU admission	60(27.4)	27(22.5)	0.324^a^
Length of stay after CD	8(7–9)	7(7–8)	< 0.001^b^

Data are presented as median (interquartile range) or n (percentage)

RBC, red blood cell; FFP, fresh-frozen plasma; ICU, intensive care unit

^a^ Chi-square test; ^b^ Wilcoxon rank-sum test

## Discussion

The finding that 35.4% of cases of placenta accreta spectrum (PAS) were not suspected before delivery over a 10-year period emphasizes the necessity for further improvement in PAS diagnosis. However, an examination of annual changes reveals a gradual increase in the diagnostic rate of PAS across the three hospitals, rising from 58.6 to 70.6%. This increase can be attributed to the growing comprehension of PAS among medical professionals and advancements in ultrasound diagnostic technology.

Multiple gravidities, previous cesarean deliveries, and placenta previa are well-established risk factors for the occurrence of PAS. Our findings demonstrated that the sPAS group had a higher proportion of gravidity > 3, history of previous CD, and placenta previa. Multivariate analysis indicated that these factors made PAS more easily detected prenatally. Studies have shown that when PAS is complicated by placenta previa and a history of previous cesarean delivery, the prenatal diagnosis rate can reach 95-98% with prenatal ultrasonography and/or MRI scans [15, 16]. However, from another perspective, when PAS cases are atypical, meaning that only some or none of these major known risk factors are present, does the prenatal suspicion rate decrease? Our results confirm this conjecture, as cases with fewer risk factors for PAS have a lower prenatal diagnosis rate. In fact, when none of these three risk factors were present, all 11 PAS cases were not identified prenatally. More detailed data in Table S2 revealed that when only one of the high-risk factors mentioned above was present, the prenatal diagnosis rate of PAS with the risk factor of gravidity > 3 was significantly lower than the other two high-risk factors (0% vs. 47.1% vs. 48.8%). Furthermore, in cases where both high-risk factors were present, the prenatal diagnosis rate of PAS cases with the risk factors of gravidity > 3 and a history of previous cesarean delivery was also lower than the other two combinations (42.1% vs. 66.7% vs. 66.7%). This observation suggests that PAS cases combined with a history of previous cesarean delivery and/or placenta previa are more likely to receive attention from clinicians. Our study’s results align with another research study, which showed that when PAS pregnant women were not complicated by placenta previa, the prenatal diagnosis rate significantly decreased (87% vs. 38%) [17].

Limited research has been conducted on the association between a history of intrauterine adhesions or placenta previa and PAS. Furthermore, studies examining the relationship between a history of clinically confirmed PAS and recurrent PAS have yielded inconsistent conclusions [18–20]. Consequently, clinicians may overlook the significance of the aforementioned histories in relation to PAS. However, recent studies have demonstrated that these histories can serve as potential risk factors for PAS. Tavcar et al. discovered that the rate of PAS in pregnancies following hysteroscopic treatment of Asherman’s syndrome was 23.7%, significantly surpassing the average incidence rate of PAS [21]. Additionally, a separate study revealed that irrespective of the mode of previous delivery (vaginal or CD), women with a history of placenta previa exhibited a significantly higher rate of PAS compared to the control group [22]. These two studies indicate that these histories can potentially serve as risk factors for PAS in subsequent pregnancies. What’s more, Tavcar’s study further revealed that prenatal diagnosis was only made in 14.3% of PAS cases (3/23), highlighting the lack of dependable clinical predictors and clinical awareness as potential factors contributing to this low diagnosis rate.

Despite limited research on the relationship between placental location and PAS [12, 23, 24], both these studies and our findings demonstrate a significantly lower prenatal diagnosis rate for posterior wall PAS compared to anterior wall PAS. This difference may be attributed to various factors. Firstly, the diagnosis of PAS is primarily based on observing the morphology of the placenta and the blood flow between the placenta and uterus using ultrasonography. Detecting PAS becomes challenging when ultrasound needs to penetrate the fetal body. Additionally, research results indicate that posterior wall PAS often exhibits shallower placental invasion and a smaller invasion area. This leads to less obvious ultrasonographic signs and an increased likelihood of going undetected [24]. According to a recent meta-analysis, the prenatal diagnosis rate of ultrasound for posterior wall PAS was only 52.4%, with MRI showing a slightly higher rate of 73.5%. However, both rates were lower than those for anterior wall PAS [23]. Ultrasound does not appear to significantly enhance diagnostic confidence for posterior wall PAS, particularly in less invasive cases, whereas MRI may be more beneficial. Despite the limited research on this topic, further cohort studies are necessary.

Whether antenatal suspicion of PAS can lead to better maternal outcomes is controversial [5, 6, 25]. Our findings indicate that despite higher rates of invasive cases and abdominal and pelvic organ injuries in the sPAS group, the estimated blood loss and need for blood product transfusion within 24 h were significantly lower compared to the uPAS group, suggesting a more favorable maternal outcome in the sPAS group. These results align with a recent study that employed a standardized multidisciplinary approach for sPAS cases [6]. Despite the lack of uniformity in approach among the three hospitals included in our study, appropriate measures were taken prior to surgery for suspected PAS cases, such as thorough examinations (ultrasonography or MRI), scheduled cesarean delivery, provision of a blood bank, placement of an abdominal aorta balloon block, and timely hysterectomy. These measures have demonstrated effectiveness in reducing intraoperative and postoperative bleeding. Our study, along with Erfani’s study, suggests that a multidisciplinary approach may enhance the maternal outcome of PAS. However, due to the relatively small sample sizes in both studies, further research involving a larger population is necessary to validate its reliability. Furthermore, it is imperative to mobilize sufficient medical resources to enhance the maternal outcomes of PAS cases that cannot be diagnosed before delivery.

A significant advantage of this study is the inclusion of pathological reports for all cases, resulting in more accurate diagnosis and grading of PAS while minimizing false positives and bias. Additionally, through comprehensive medical record abstraction, we were able to identify factors that are often overlooked.

However, our study does have certain limitations. Firstly, the most significant limitation is that this study is retrospective, leading to a higher proportion of invasive cases in the sPAS group. These severe cases may have been more readily diagnosed during pregnancy, potentially introducing bias into our results. Additionally, in order to minimize misclassification, we excluded more cases that underwent conservative management and could not provide pathological results. However, we assert that cases necessitating hysterectomy hold greater significance than those that can be managed conservatively. Lastly, to ensure an adequate sample size, we conducted a retrospective review of medical records spanning the past 10 years. While this approach has strengths, it is also a limitation as imaging and surgical skills may have advanced during this period.

## Conclusions

In conclusion, our data revealed that approximately one-third of PAS cases were not suspected during antenatal screening, and these unsuspected cases exhibited poorer outcomes compared to the suspected cases. Risk factors for lack of suspicion include a history of intrauterine adhesions, clinically confirmed PAS, posterior placenta, and placenta previa. Increasing awareness of these risk factors could facilitate early diagnosis of PAS and enhance maternal outcomes.

Subsequent studies should assess whether the uPAS-related risk factors identified in our study can enhance the rate of prenatal PAS detection, thereby optimizing prenatal management and outcomes. Furthermore, it is crucial to identify more dependable prenatal diagnostic markers or modify existing diagnostic approaches in order to enhance the prenatal diagnosis rate of atypical PAS. Furthermore, larger and more comprehensive collaborative studies are necessary to investigate the relationship between a multidisciplinary management protocol and improved peripartum outcomes in China.

### Electronic supplementary material

Below is the link to the electronic supplementary material.

Supplementary Material 1

## Data Availability

The datasets used and/or analysed during the current study are available from the corresponding author on reasonable request.

## Abbreviations

PAS: Placenta accreta spectrum
CD: Cesarean delivery
