# Supplementary material for: Incidence, risk factors and maternal outcomes of unsuspected placenta accreta spectrum disorders: a retrospective cohort study
Source: BMC Pregnancy Childbirth. 2024 Jan 23;24:76. doi: 10.1186/s12884-024-06254-z (PMC10804779; doi:10.1186/s12884-024-06254-z)
Supplement: Supplementary file 1 — Supplementary Material 1 [file 12884_2024_6254_MOESM1_ESM.docx]

**Table S1** Number of PAS cases and rate of suspicion classified by year.

| **Year** | **All PAS** | **sPAS** | **uPAS** | **Percentage of antenatal suspicion** |
| --- | --- | --- | --- | --- |
| 2013 | 29 | 17 | 12 | 58.6% |
| 2014 | 30 | 19 | 11 | 63.3% |
| 2015 | 35 | 22 | 13 | 62.9% |
| 2016 | 46 | 28 | 18 | 60.9% |
| 2017 | 52 | 32 | 20 | 61.5% |
| 2018 | 40 | 26 | 14 | 65.0% |
| 2019 | 39 | 26 | 13 | 66.7% |
| 2020 | 32 | 24 | 8 | 75.0% |
| 2021 | 19 | 13 | 6 | 68.4% |
| 2022 | 17 | 12 | 5 | 70.6% |

PAS, placenta accreta spectrum.

**Table S2** Rate of suspicion classified by the number of major known risk factors.

|  | **sPAS（219）** | **uPAS（120）** | **Percentage of antenatal suspicion** |
| --- | --- | --- | --- |
| None of them | 0 | 11 | 0.0% |
| Only one risk factor | 28 | 34 | 45.2% |
| *Only gravidity >3* | 0 | 4 | 0.0% |
| *Only history of previous CD* | 8 | 9 | 47.1% |
| *Only placenta previa* | 20 | 21 | 48.8% |
| Two risk factors | 72 | 43 | 62.6% |
| *Gravidity >3 + history of previous CD* | 8 | 11 | 42.1% |
| *Gravidity >3 + placenta previa* | 10 | 5 | 66.7% |
| *History of previous CD + placenta previa* | 54 | 27 | 66.7% |
| All the three factors | 119 | 32 | 78.8% |

Risk factors: gravidity>3, history of previous CD (number of CD≥1) and placenta previa.
